# Episodic Behavioural Regression in an 8-Year-Old Female: Sequelae of 22q11.2 Duplication Syndrome

**DOI:** 10.1155/2018/1394356

**Published:** 2018-08-09

**Authors:** A. Bahji, S. Khalid-Khan

**Affiliations:** Division of Child & Youth Psychiatry, Queen's University, Canada

## Abstract

22q11.2 duplication syndrome is a recently discovered genetic syndrome with unclear neuropsychiatric sequelae. While its connection to 22q11.2 deletion syndrome is actively investigated, case reports on the neuropsychiatric sequelae of affected individuals have been previously described, largely focusing on comorbid autism spectrum disorder. Here, we present the case of an 8-year-old female experiencing episodes of severe behavioural regression following medical illness. We analyze the case and relate it to the available literature and identify potential risk factors.

## 1. Introduction

Since its recent discovery in 1999 [1], 22q11.2 duplication syndrome has been found to be associated with a highly variable neuropsychiatric phenotype, including learning disability [2], intellectual disability (ID), and, most recently, autism spectrum disorder (ASD) [3–5]. The most common clinical features of 22q11.2 duplication syndrome include congenital heart, craniofacial, and neurological abnormalities, such as feeding and sensory and motor problems [6].

Of note, ASD has received recent attention in 22q11 duplication syndrome and has been a frequently described neuropsychiatric sequelae of several other types of genetic anomalies, including trisomy 22 and 22q13.3 deletion [4, 7–10]. A recent article comprehensively reviewed the role of copy number variants at the 22q11.2 locus and its association with ID and ASD [5]. In a previous report, a 9-year-old female was referred for behaviour and language difficulties and ultimately diagnosed with autism; on physical exam, she was found to have craniofacial and cardiac anomalies typical of velocardiofacial syndrome, but cytogenic analysis revealed a 22q11.2 duplication, rather than deletion [9]. In addition to ASD, symptoms of attention deficit hyperactivity disorder (ADHD), learning disability, speech difficulties, and aggression have also been described in 22q11.2 duplication syndrome [9].

To date, research describing the clinical presentation of affected individuals has been mostly limited to case reports [11], and research on other types of presentations has been more limited. In this report, we describe an interesting sequela of a 22q11.2 duplication carrier that has not been previously reported. This patient developed episodic aggression and increased social anxiety, and academic and social declines following physical illnesses; this case highlights the possibility that physical stress may elicit psychiatric symptoms in 22q11.2 duplication patients.

## 2. Case Presentation

Our patient was an 8-year-old Caucasian female referred to our neurodevelopmental disorders clinic following periods of extreme behavioural problems in the context of physical illness. On family history, her maternal grandfather and two maternal first-cousins were reported to have been diagnosed with fragile X syndrome, while her mother and two maternal aunts were reported to be carriers for the fragile X premutation; however, the family was unable to provide additional details on the extent of the fragile X diagnoses. On her father's side, there were several family members with identified learning disabilities. There was no other significant family history of psychiatric or medical illness.

Prenatal, birth, and developmental history were unremarkable. The patient was described as an “easy” baby. There was no ongoing conflict described between the parents. She was described as always being a good student, active in many hobbies, and well-adapted socially. Her past medical history was significant for a diagnosis of ADHD, which had been made by the pediatrician two years before the onset of her behavioural symptoms. The patient's comorbid ADHD had been previously treated with methylphenidate, lisdexamfetamine; however, the medications were discontinued after the patient's behavioural syndrome surfaced without any clear benefit. At the time of assessment, the patient was taking guanfacine. There was no history of head trauma. There was no other significant past psychiatric history.

The active symptoms and signs reported by the patient and her family included aggression, enuresis, increased social anxiety symptoms, fearfulness and increased dependence on caregivers, academic decline (in terms of grades and attendance at school), and social decline (less interested in interactions with family and peers). The patient's parents described her behaviour to have “regressed,” which included social withdrawal from family and peer gathering but also many times when the patient was found to be “hiding behind the chair.” The first of these episodes occurred a few days after she had developed bacterial pneumonia. Other episodes occurred shortly after she had developed streptococcal sore throat and chicken pox. The only other preceding event our patient and her family were able to identify was that they had been travelling a few days before the development of the first episode of behaviour problems.

The abovementioned behavioural syndrome was initially accompanied by a sense of anxiety; however there was an absence of obvious physical symptoms or signs (such as palpitations, shortness of breath, tightness in the chest, and numbness in the arms). Subsequently, the syndrome subsided a few weeks after the physical illness had resolved, and our patient was described as having “returned to her baseline” by her parents. There was no evidence of psychosis during these episodes.

The physical exam, performed by a pediatrician and subsequently repeated by the patient's family physician, was entirely “unremarkable,” including a normal full neurological and thyroid exam. Screening medical investigations, including a complete blood count, renal function, liver function, and thyroid panel, were noncontributory. Pediatric autoimmune neuropsychiatric syndrome was also considered, given her exposure to streptococcus infection. Upon referral to the genetics clinic, cytogenetic analysis was performed, which was significant for a 22q11.2 microduplication. Genetic testing was unremarkable for additional microdeletions or microduplications and was negative for the fragile X premutation.

When we had assessed our patient, she appeared stable and was doing well overall. She was attending school regularly and doing well academically. She also had been engaging in a variety of extracurricular activities and had established a secure social network outside of her immediate family. Her parents described her to be doing well both socially and emotionally at home. We primarily diagnosed our patient with ADHD based on history and an unspecified behavioural syndrome that was related to physical illness. We also noted the presence of residual social and generalized anxiety symptoms and recommended a referral to a cognitive behavioural therapy group for skill building. She followed up with her pediatrician a few months later and has been reportedly doing well. To date, she has had no further episodes and has declined further care.

## 3. Discussion

Although there are overlapping features with 22q11.2 microdeletion syndrome [12, 13], the neuropsychiatric profile of 22q11 duplication has been comparatively little investigated. However, there are preliminary reports that the two syndromes may be more different than alike, with recent evidence suggesting that the presence of 22q11 duplication may in fact be protective against schizophrenia, while the deletion is associated with an increased risk of psychosis [14].

While behavioural problems are not uncommon in individuals with genetic disorders, they are less likely to present as episodic and their symptom onset tends to be associated with psychosocial, rather than physiological stressors. As such, cases of severe behavioural regression, like the ones described by our patient, have not been previously reported in individuals with 22q11 duplication [7]. In addition, patients with genetic syndromes are more likely to receive a diagnosis of comorbid neurodevelopmental disorder, such as autism; however, our patient had been previously diagnosed with ADHD. More recently, it appears that the 22q11.2 region has been associated also with increased rates of ADHD, so this finding may be consistent with emerging research [15].

At a molecular level, 22q11.2 duplication has been studied in mouse models, and affected mice have demonstrated delays in working memory development [16, 17] as well as clozapine-responsive behavioural abnormalities [18]. While 22q11.2 duplication has been considered a protective factor for psychosis [14], cases of psychosis have recently been reported [19, 20]. In a previous study, cognitive decline was shown to signal the later onset of psychosis in 22q11.2 deletion carriers [21]; however, the psychosis potential in 22q11 duplication has been less well described and may be worrisome for future psychosis.

This case stands out based on the prolonged duration of the first episode of behavioural regression despite resolution of the physical illness, as well as the remarkable family history of genetic disorder. Our patient is also remarkable in that she did not have any residual behavioural signs when she was physically well.

Thus, severe episodic behavioural regression following physical illness in individuals with genetic disorders, like 22q11 duplication, seems associated with an elevated vulnerability to psychosocial or physiological stressors. A family history of genetic syndromes may be another risk factor. However, it remains unclear as to why these specific behavioural problems follow an episodic pattern, as in the case of our patient. Moreover, this case highlights the possibility that physical stress may unmask underlying psychiatric conditions in affected individuals.

## 4. Conclusion

We presented the case of an 8-year-old female who experienced several episodes of behavioural regression after physical illnesses. It is proposed that genetic disorders in young individuals with risk factors such as a family history of other genetic problems, like fragile X syndrome, and a state of acute or chronic distress (either psychological or physiological) increase the risk of severe behavioural problems; these can recur for several weeks after the resolution of the stressor. Little is known of the neurophysiology involved and further investigations are needed to elucidate the connection between behavioural regression, physical stress, and 22q11 pathology.
